# Uncovering New Horizons: Update to Quadruple-D Score to Predict Stone-Free Rate with Advanced Non-invasive Lithotripsy Technology

**DOI:** 10.5152/tud.2025.24152

**Published:** 2025-05-21

**Authors:** Aaron Tigor Sihombing, Zola Wijayanti, Steven Steven, Dicky Stefanus

**Affiliations:** Department of Urology, Hasan Sadikin Academic Medical Center, University of Padjadjaran, Bandung, Indonesia

**Keywords:** Nomogram, piezoelectric, shockwave lithotripsy, stone free-rate

## Abstract

**Objective:**

To evaluate the efficacy of the Quadruple-D scoring system in predicting stone-free rate (SFR) using the newer Generation Piezoelectric lithotripter.

**Methods:**

A prospective observational study was conducted from January to December 2023, involving patients who underwent extracorporeal shock wave lithotripsy (ESWL) for renal stones sized 5-20 mm. Evaluation parameters included stone density, skin-to-stone distance (SSD), stone size (ellipsoid stone volume), and location, with Quadruple-D scores calculated based on predetermined cutoffs. Extracorporeal shock wave lithotripsy sessions utilized the “Piezolith 3000 Plus” lithotripter, and SFR was assessed 4 weeks post-procedure. Statistical analysis included Student’s *t*-test and receiver operating characteristic curve analysis.

**Results:**

Of the 40 eligible patients, 75% achieved stone-free status post-ESWL. Stone density and SSD emerged as leading predictors of SFR, with new cut-off values identified. Comparative analysis demonstrated improved predictive power of the Piezolith Q-D score over the previous Quadruple-D score (AUC: 84% and 80% respectively). Although stone location and size also influenced outcomes, their significance varied in this study.

**Conclusion:**

The Piezolith Q-D score system exhibits promise in predicting SFR post-ESWL with piezoelectric lithotripters. External validation and larger-scale studies are warranted to establish the scoring system’s reliability and applicability across diverse populations.

**Main Points**

- The Piezolith Q-D score, an updated version of the Quadruple-D score, was developed to predict stone-free rates after ESWL using a piezoelectric lithotripter.
- Stone density (HU) and skin-to-stone distance (SSD) were the most significant predictors of ESWL success, with newly identified cutoff values of 1201.5 HU and 12.54 cm, respectively.
  The Piezolith Q-D score showed better predictive accuracy (AUC 0.842) than the original Quadruple-D score (AUC 0.803) in determining post-ESWL stone-free status.
- Stone size (ellipsoid volume) was moderately associated with outcomes, while stone location showed no significant impact on ESWL success in this study.

## Introduction

In the last half-century, the management of kidney stones has continued to evolve, starting from open surgery to non-invasive methods such as extracorporeal shock wave lithotripsy (ESWL) and minimally invasive techniques such as percutaneous nephrolithotomy (PCNL) and retrograde intra-renal surgery (RIRS). The advancement of this technology has significantly favored minimal and non-invasive procedures as the primary choice due to their relatively lower complication rates compared to open surgery.^1^ Extracorporeal shock wave lithotripsy remains a part of the armamentarium for managing kidney stones smaller than 20 mm and is favored by patients for its non-invasive nature, while for urologists, ESWL procedures are relatively simple.

Currently, there are 3 types of ESWL lithotripters: electrohydraulic (EH), electromagnetic (EM), and piezoelectric. Research has shown that piezoelectric, EH, and EM lithotripters are equally effective and have similar safety profiles for kidney stones smaller than 2 cm, but with various stone-free rates (SFRs): 67.88%, 70.01%, and 51.22%, respectively.^2^^,^^3^ The difference in SFRs among the various generator types is influenced by the ESWL machine’s ability to focus shock waves, as well as the energy and shock wave frequency of the ESWL generator.^3^^,^^4^ Advantage of piezoelectric machines is their better focusing ability with a smaller tissue damage radius compared to EH, thus allowing for ESWL procedures without anesthesia.^3^^,^^5^ Even the latest generation of piezoelectric ESWL machines is promised to have wave penetration power of up to 165-200 mm and equipped with triple focus, making them considered more effective in breaking down stones of all shapes compared to their predecessors.^6^

To date, many factors believed to affect the SFR after ESWL, such as stone location, size, composition, Hounsfield units (HU), and skin-to-stone distance (SSD). In 2015, Tran et al^7^ created the Triple-D scoring system (stone dimension, distance, density), which was later improved by Ichiyanagi et al^8^ into the Quadruple-D scoring system (stone dimension, distance, density, and distribution). However, these scoring components were tested on EH ESWL machines and have not yet been tested on piezoelectric ESWL machines, which are widely used today. The aim of this study is to evaluate the efficiency of this Quadruple-D scoring system in predicting SFR in patients with kidney stones <20 mm treated with the ESWL Piezolith lithotripter.

## Material and Methods

This is a prospective observational study conducted from January to December 2023 at a tertiary hospital in Indonesia. Although approximately 38 patients are needed to achieve significant statistical value, in this study, it was decided to include all patients undergoing ESWL procedures based on indications from guideline.^9^^,^^10^ The study was conducted following the guidelines set by the Declaration of Helsinki. The research protocol was approved by the research ethics committee of Dr. Hasan Sadikin General Hospital Bandung Date: 5th June 2023 Approval No: LB.02.01/X.6.5/202/2023. Written consent was signed by all the participants. The inclusion criteria used were adult patients (over 18 years old) undergoing ESWL for single stones measuring 5-20 mm, as assessed by non-contrast urology computed tomography scans (NCCT). The exclusion criteria for this study included: (1) pregnant patients, (2) patients with active urinary tract infections, (3) patients with uncorrected coagulation disorders, (4) patients with abnormal kidney structures, (5) patients unable to complete ESWL sessions according to the prescribed energy and shock wave numbers, (6) patients with a body mass index (BMI) over 30 because low-dose NCCT was used, and (7) patients with a history of previous ESWL on the particular kidney side (not residual stone).

Before ESWL procedures, all candidates underwent assessment with NCCT to evaluate parameters for scoring, including HU, SSD, stone size, and stone location. Ellipsoid stone volume (ESV) was calculated using the formula π/6 × (anteroposterior stone size × transverse × craniocaudal) in millimeters.^8^ Skin-to-stone distance was the average distance from the body surface to the stone center at 0°, 45°, and 90° angles assessed on axial NCCT slices. The Quadruple-D score was calculated by summing up components based on the cutoffs established by Tran et al,^7^ namely ESV < 150 µL, SSD < 12 cm, HU < 600, and stone location whether in the inferior calyx or not. The unfavorable parameter was scored 1; otherwise, it scored 0. The total score ranged from 0 to 4 (worst to best).^11 ^

This study used the Piezoelectric lithotripter, Wolf Piezolith 3000 Plus. The ESWL procedures were conducted with shock wave frequencies ranging from 30 to 90 waves per minute, with a total of 3500-4000 shocks per therapy session, and energy levels ranging from 1 to 15 mJ. Data were collected from all patients after the first ESWL session and evaluated 4 weeks later to assess SFR by performing another non-contrast CT scan.

Statistical calculations were performed using Student’s *t*-test for numeric variables and chi-square for categorical variables to assess significant differences in each parameter. Receiver operating characteristic (ROC) curves were formed to calculate new cutoff values, which were then compared with existing cutoffs using the area under the curve (AUC).

## Results

Seventy-two patients underwent ESWL therapy from January to December 2023, with 40 patients meeting the inclusion and exclusion criteria for this study. Thirty patients (75%) were included in the stone-free group during follow-up. There were no significant differences found in age, gender, or stone location between the stone-free and non-stone-free groups (Table 1).

The mean ESV in the stone-free group was 420.62 ± 374.01, while in the stone residue group it was 597.69 ± 460.31 (*P *= .022). Stone density, assessed by HU values from NCCT, had mean values in the stone-free group of 1054.17 ± 157.557 and in the stone residue group of 1345.60 ± 145.194 (*P* = .000). The third parameter was SSD, with mean SSD in the stone-free group at 11.25 ± 1.46 cm and in the residual stone group at 13.29 ± 0.74 (*P* = .000) (Table 1).

**Figure 1.**
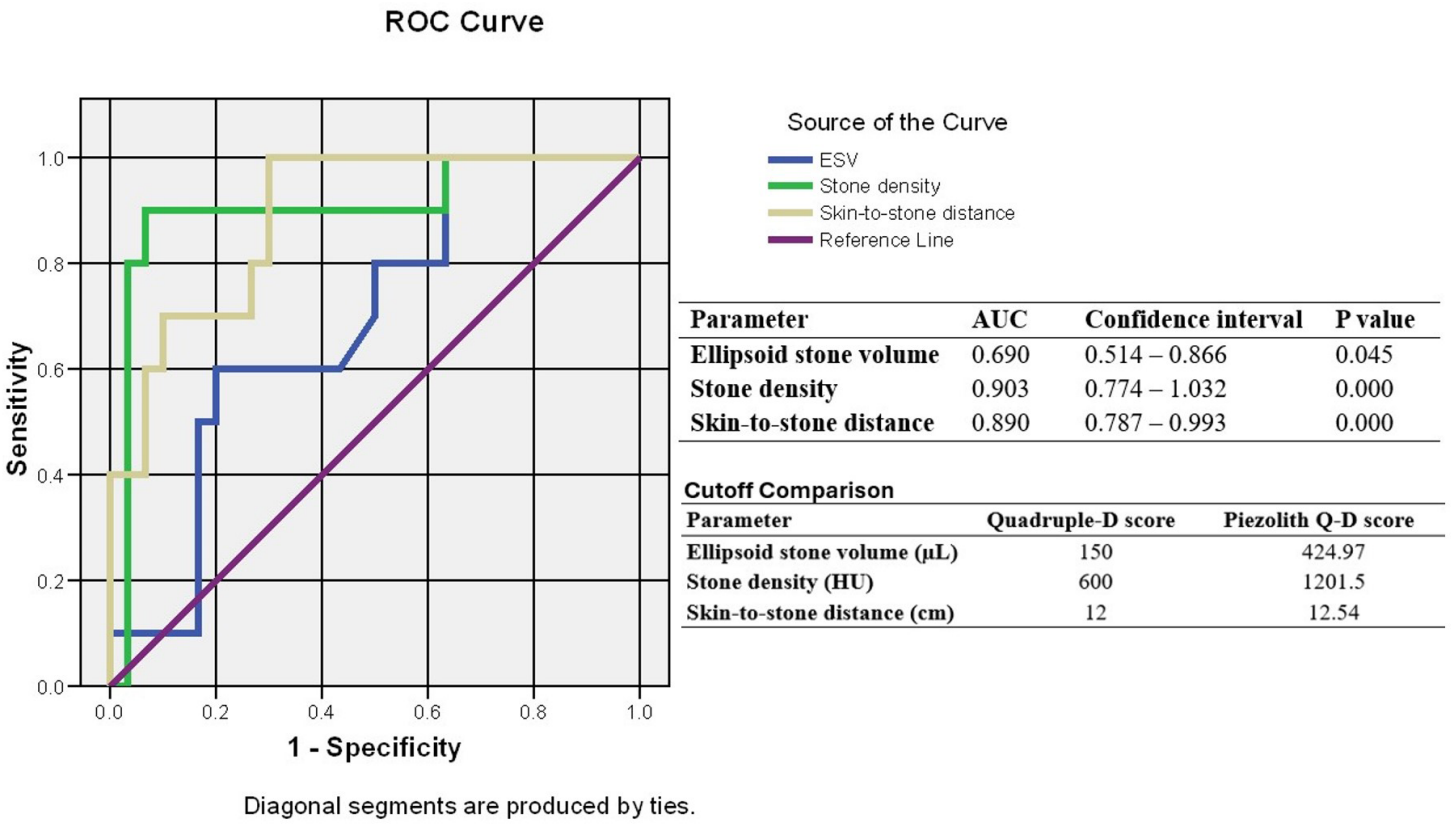
ROC curve for Piezolith Q-D score parameter.

Furthermore, AUC calculations for ESV, stone density, and SSD were 0.690, 0.903, and 0.890, respectively. The cutoff values for each variable to achieve the best sensitivity and specificity were 424.97 µL, 1201.5, and 12.54 cm for ESV, stone density, and SSD respectively (Figure 1).

New Quadruple-D score calculations were based on parameters found in this study, which later called the Piezolith Q-D score, with results ranging from 0 to 4. Stone-free rates were found to be 100%, 93.3%, 75.0%, 50%, and 25% for each score. When using parameters from initial Quadruple-D studies, stone-free rates were found to be 100%, 100%, 75%, and 45.5%. The median Piezolith Q-D score in the stone-free group was 1, while with the Quadruple-D score it was 3 (Table 2).

**Figure 2.**
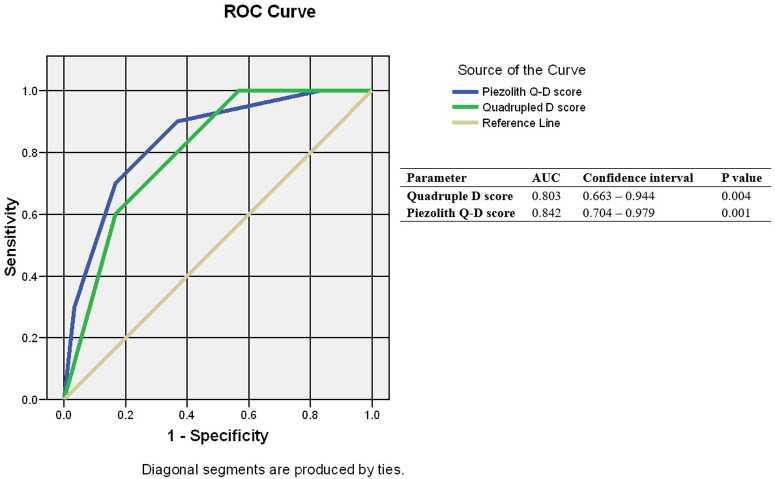
ROC curve for scoring comparison.

Prognostic testing was conducted using ROC curves, with the AUC score for the previous score being 0.803 with a 95% CI of 0.663-0.944, while the AUC for the Piezolith Q-D score was 0.842 with a 95% CI of 0.704-0.979 (*P* = .001) (Figure 2). Based on this analysis, it can be said that the Piezolith Q-D score system is better able to predict SFR after ESWL with piezoelectric machines compared to the existing Quadruple D score.

## Discussion

Nowadays, ESWL has been used successfully as an outpatient procedure for the treatment of urinary tract stones with minimal morbidity. Shockwave lithotripsy has many advantages over RIRS and PCNL, such as lesser complication rates, shorter hospital stays, and ease of use by surgeon. However, the SFR after ESWL varies from 35% to 88% in different studies, regardless of the position and composition of the stone, which are become the indicators for ESWL success.^12^ This variety indicates that many factors affect the outcome, and decision-making for patient selection is not simple. With the aim of predicting post-ESWL outcomes and guide to choose appropriate patients to undergo the procedure, nomograms were developed.^13^

A nomogram provides several advantages, such as minimize re-treatment and reduce economic burden on the healthcare system. Several nomograms have been suggested by previous authors, such as Triple D score, Kim JK, and S3HoCKwave score, which were published in 2015, 2016, and 2019 respectively.^7^^,^^14^^,^^15^ The Triple D score has been updated to the Quadruple D score by Ichiyanagi O et al^8^ with an additional parameter.

In this study, the Quadruple-D score is tried to be evaluated to predict the SFR in the ESWL procedure. The main advantage of this score is that it is easy to calculate and can be used in routine radiology reports to aid physician in the clinical decision-making process of treatment kidney stone with size less than 20 mm.

In this study, stone density and SSD are leading independent predictor factors on SFR after ESWL. Several studies showed that the energy of the shockwave and the shock amount needed for fragmentation were related to these 2 factors. A study by Wang et al^16^ showed that stone density of more than 900 has a poor prognosis for ESWL. Park et al^17^ and Ouzaid et al^18^ found the cut-off point for high SFR to be 863 and 970 respectively. The cut-off point suggested by previous Triple-D and Quadruple-D scores was 600.^7^ In this study, it was found that the cut-off point for HU was 1201.5, which is higher than any stone density limit stated in previous studies. This difference in the cut-off point for stone density is due to the superior focusing capabilities of piezoelectric devices; the stone fragmentation process is more effective and efficient.

Secondly, SSD also plays a role in determining the stone-free rate. The national and international guidelines state that one favorable factor for ESWL is an SSD of less than 10 cm.^19^ In this study, it was found that the cut-off point for SSD to be 12.54 cm, which is almost similar to a study conducted by Timothy et al which was 12 cm.^7^ This value is higher compared to other studies conducted by Pareek et al,^20^ who found that the threshold for the success of ESWL is 10 cm. Research by Wiesenthal et al^21^ established that an SSD value > 11 cm results in significantly worse success compared to < 11 cm. Previously, BMI was considered an important parameter in predicting ESWL success, but several authors have concluded that SSD holds more value due to variations in body type and body composition such as muscle, fat, and water among individuals and races.^13^ The differences in the SSD threshold among various studies may be attributed to the heterogeneity of measurement methods of SSD from different angles.^22^ Additionally, the operator’s skill level and advancements in lithotripter technology could also be contributing factors to these differences. Most existing studies predominantly utilize ESWL devices with EH lithotripter types, whereas in this study, a new generation piezoelectric device, namely the Piezolith 3000 plus was employed, which offers greater penetration power and features triple focus capability, allowing for improved shot accuracy compared to previous generations.^6^

Stone location was also stated as an independent predictor for ESWL outcome. Stones located in the renal pelvis and superior or medial calyx had a higher ESWL success rate than those in the inferior calyx, which were consistent with several previous studies.^8^^,^^23^^,^^24^ In this study, kidney stone location is not significantly different in determining the outcome of ESWL. The findings is similar to study by Al-Zubi et al,^25^ that found stone location has no significant effect on the ESWL response rate. Multicenter studies with larger study populations are needed to confirm this outcome.

Lastly, stone size, which can be represented by the ESV, has a well-known negative correlation between stone size and stone-free rate after ESWL. Stone size is universally evaluated by measuring a stone’s maximum length or surface area, but this can be problematic because renal stones are usually are irregular with 3 dimension complex geometric properties.^13^ A study conducted by Bandi et al,^26^ found no significant correlation between stone size measured by maximum length only and ESWL success. However, there was significant lower volume in patients who achieved SFR after ESWL, as confirmed by study by El-Nahas.^27^ In this study, ESV is significantly different between groups, with cutoff volume of 424.97 µL having the highest prognostic value to determine stone-free status.

While simultaneous consideration of CT-based metric parameters may pose challenges, combined analysis of preoperative factors can enhance predictive power and facilitate the application of these tools in clinical practice. In this study, a new cutoff point for the Quadruple-D score was generated using a Piezoelectric lithotripter that differed from previous EH and EM devices. This study also has several limitations. Firstly, it was conducted in 1 center and performed with a single Piezoelectric model device, which may not represent some other populations. Secondly, the study limited the patient BMI to under 30 due to the limitation of the low-dose NCCT. Lastly, the sample size was smaller than the previous nomogram study.

In the population with renal stones between 5 and 20 mm, the Piezolith Q-D score system can be used as a guide to clinically decide candidates for ESWL. Further external validation of this scoring is needed to confirm the validity and reliability of the scoring system.

## Figures and Tables

**Table 1.** Comparison of Study Population and Scoring System Parameters Result

Parameter	Group Study		*P*
	Stone Free (n = 30)	Stone Residue (n = 10)	
+**Demographic** **Comparison**			
Age (years) Mean ± SD	52.60 ± 10.40	55.90 ± 9.45	.380*
Gender Male Female	12 (70.6%) 18 (78.3%)	5 (29.4%) 5 (21.7%)	.580¶
Stone location Inferior calyx Other	18 (75.0%) 12 (75.0%)	6 (25.0%) 4 (25.0%)	1.000¶
+**Scoring Component Comparison**			
Ellipsoid stone volume (µL)	420.62 ± 374.01	597.69 ± 460.31	.022*
Stone density (HU)	1054.17 ± 157.557	1345.60 ± 145.194	.000*
Skin-to-stone distance (cm)	11.25 ± 1.46	13.29 ± 0.74	.000*

(*) compared using student *t*-test.

(¶) compared using Chi square analysis.

**Table 2.** Total Score Comparison

Parameter	Group Study		*P*
	Stone Free (n = 30)	Stone Residue (n = 10)	
Quadruple D score Score 0 Score 1 Score 2 Score 3 Score 4	0 2 (6.7%) 11 (36.7%) 12 (40.0%) 5 (45.5%)	0 0 0 4 (40.0%) 6 (60.0%)	.024
Piezolith Q-D score Score 0 Score 1 Score 2 Score 3 Score 4	5 (16.7%) 14 (46.7%) 6 (20.0%) 4 (13.3%) 1 (3.3%)	0 1 (10.0%) 2 (20.0%) 4 (40.0%) 3 (30.0%)	.015

Compared using Chi square analysis.

## Data Availability

The data that support the findings of this study are available upon request from the corresponding author.
